# Proteomic Analysis Identifies Potential Markers for Chicken Primary Follicle Development

**DOI:** 10.3390/ani11041108

**Published:** 2021-04-13

**Authors:** Armughan Ahmed Wadood, Jingyuan Wang, Liping Pu, Qaisar Shahzad, Muhammad Waqas, Xingting Liu, Long Xie, Lintian Yu, Dongyang Chen, Rana Waseem Akhtar, Yangqing Lu

**Affiliations:** 1 State Key Laboratory for Conservation and Utilization of Subtropical Agro-Bioresources, College of Animal Science and Technology, Guangxi University, Nanning 530000, China; armughanwadood@gmail.com (A.A.W.); 15738380138@163.com (J.W.); 18775352516@163.com (L.P.); raoqaisarshahzad@gmail.com (Q.S.); waqas_sk@yahoo.com (M.W.); xtliu@gxu.edu.cn (X.L.); mrchildren77582580@163.com (L.X.); ylt254523499@163.com (L.Y.); Alexzander110@outlook.com (D.C.); 2 Department of Veterinary and Animal Sciences, Muhammad Nawaz Sharif University of Agriculture, Multan 61000, Pakistan; dr.ranawasim2@gmail.com

**Keywords:** chicken, small and developing primary follicles, proteomics, differentially expressed proteins

## Abstract

**Simple Summary:**

Our study presents a comprehensive approach elaborating the mechanism of primary follicle development in the chicken. The identified differentially expressed proteins of small and developing primary follicles (SPFs and DPFs) could be used as potential markers in chicken primary follicle development. The DEPs have their functional involvement in different processes including glycolysis, pyruvate metabolism, amino acid synthesis, and oocyte meiosis. The *Anxa2*, *Pdia3,* and *Capzb* have a connotation in primary follicle development. These findings were validated by real-time quantitative PCR and provided a basis for the exploration of DEPs as suitable makers related to the primary follicle development in chicken.

**Abstract:**

Follicles’ development in chicken imparts a major impact on egg production. To enhance the egg-laying efficiency, comprehensive knowledge of different phases of follicular development is a prerequisite. Therefore, we used the tandem mass tag (TMT) based proteomic approach to find the genes involved in the primary follicular development of chicken. The primary follicles were divided into two groups—small primary follicles (81–150 μm) and developed primary follicles (300–500 μm). Differential expression analysis (fold change > 1.2, *p*-value < 0.05) revealed a total of 70 differentially expressed proteins (DEPs), of which 38 were upregulated and 32 were downregulated. Gene ontology (GO) enrichment analysis disclosed that DEPs were intricate with cellular protein localization, the establishment of protein localization, and nucleoside phosphate-binding activities. Kyoto Encyclopedia of Genes and Genomes (KEGG) enrichment pathway indicated the involvement of DEPs in different metabolic pathways such as glycolysis, pyruvate metabolism, galactose metabolism, and fructose and mannose metabolism. The current proteomic analysis suggested suitable markers such as *Anxa2*, *Pdia3,* and *Capzb*, which may serve as a potential role for primary follicle development. The present study provides the first insight into the proteome dynamics of primary follicle development and would play a potential role for further studies in chicken to improve egg productivity.

## 1. Introduction

Eggs are an inexpensive source of high-quality protein, essential vitamins, and minerals necessary for a well-balanced diet and a healthy life. Current global per capita egg consumption estimates approach 9 kg annually but vary greatly on a regional basis. By 2050, the world’s population is expected to reach 9 billion, with the highest population growth rates occurring in regions suffering mostly from food insecurity [1]. Therefore, it is imperative to enhance egg production to fulfill the growing demand.

In avian species, the egg production is mainly vested in follicles development as these follicles had oocytes surrounded by the layers of granulosa and theca cells, intact with the oocyte plasma membrane. Follicular development involved a series of complex process or steps which ultimately results in ovulation. At the same time, thousands of developing follicles undergo atresia [1,2]. The chicken is an exclusive experimental model owning to ovarian follicles used to study follicular development [3].

Various studies have been documented the mechanism which governs follicles’ transition from prehierarchical to the ovulatory stage [4,5]. This transition phase is a highly coordinated biological process affecting different stages of follicular development including oocyte maturation, proliferation, and differentiation of granulosa and theca cells within the follicles controlled by several regulatory factors [6,7,8]. At the time of follicles selection, the candidate follicle initiates steroidogenic pathway via protein kinase A/cAMP signaling through involving steroidogenic acute regulatory protein (STAR) and hole sterol side-chain cleavage enzyme (*Cyp11a*) [9,10], while the inhibitory signals such as protein kinases retain the granulosa cells in an undifferentiated state in all follicles irrespective of the size [11].

Similarly, several proteins can also contribute to the follicle maturation process; for instance, in theca cells, the expression of annexin A2 (*Anxa2*) could induce angiogenic factors, which contribute to follicular development and eventually ovulation [12]. The matrix metalloproteinases (MMP) enzymes can intricate the follicle ovulatory process through regulating the protein disulfide isomerase A3 (*Pdia3*), which is further involved in protein folding via oxidation, reduction, and isomerization of a disulfide bond in proteins [13], and has also been involved in cell adhesion such as sperm oocyte interaction [14]. These have also been involved in cellular estradiol sequestering [15].

Recent advancement has been made in the field of chicken primordial follicle development [16,17], but very little is known about the mechanisms underlying the development of primary follicles. The atretic mechanism is the major hindrance in which all the primary follicles do not pass into the second stage, although naturally, only a few of them develop into the maturation stage [18]. Thus, increased primary follicle selection for further development can improvise the chicken egg-laying capacity. Therefore, this necessitates a comprehensive understanding of the mechanism of primary follicular development.

The proteomic methods are important molecular-level approaches used to analyze complex mechanisms that are helpful in understanding complex biological processes [19]. Furthermore, it could determine the functionally viable proteins, their properties, and mode of action [20]. The advanced proteomics procedures helped to identify the protein abundance, their differential expression, and sensitivity level. The present study aimed to investigate the molecular mechanism associated with the primary follicle involved in early development. This study also provided a comprehensive understanding of primary follicle development at the proteome level, which could pave a way for further molecular-based studies.

## 2. Materials and Methods

### 2.1. Animal Selection

**Figure 1:**
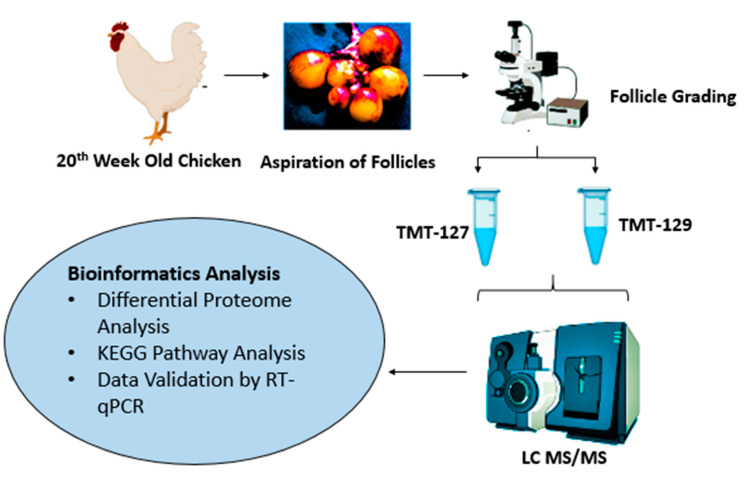
Schematic illustration of experimental design. Ovaries were isolated from 20-week-old chickens. The small follicles were aspirated and divided into two groups based on their— tandem mass tag (TMT)-127 indicates small primary follicles (81–150 μm) and TMT-129 as developing primary follicles (300–500 μm). The proteomic analysis was conducted by using Liquid chromatography–mass spectrometry (LC—MS/MS). The obtained data were analyzed for differential expression of proteins. The differentially expressed proteins (DEPSs) were subjected to gene ontology analysis and Kyoto Encyclopedia of Genes and Genomes (KEGG) pathway analysis. The proteomic data were validated by real-time quantitative PCR.

The schematic illustration of the experimental design is presented in Figure 1. The Ethics Committee constituted by the Animal Welfare Department of the Guangxi University approved the present study. Six Guangxi yellow-feather chickens, 20 weeks of age, were selected and euthanized in the study. The ovaries were removed carefully and immediately transferred in normal saline solution at 39 °C.

### 2.2. Isolation of Follicles

Ovaries were thoroughly washed using normal saline and chopped into smaller pieces of 1.5 to 2 mm in size. The chopped material was again washed with phosphate-buffered saline/polyvinyl alcohol (PBS/PVA) solution. PBS/PVA solution was prepared by mixing 1g polyvinyl alcohol (PVA) in 100 mL of phosphate-buffered saline (PBS). Primary follicles were isolated by the enzymatic method. Briefly, the chopped ovaries were placed into a Petri dish and incubated (37 °C, 5% CO_2_, 25–30 min) with Trypsin Ethylenediaminetetraacetic acid (EDTA) solution (0.25%, Sigma–Aldrich, St. Louis, MO, USA), as described previously [21,22]. The mixture of enzymes and ovaries was incubated at 37 °C in the incubator at 5% CO_2_ for 25–30 min. After digestion, all the enzymatic solution was removed by using a micropipette and washed with PBS/PVA solution to stop the digestion, and then dispersed follicles were placed into a Petri dish. Loosely attached follicles were isolated by using an insulin syringe and placed into a separate glass plate. All the isolated follicles were washed with PBS/PVA solution three times to remove debris and blood. After the isolation, follicles were graded as small primary follicles (SPFs, 81–150 μm) and developing primary follicles (DPFs, 300–500 μm), respectively.

### 2.3. Protein Extraction, Digestion, and Peptide Labeling

Follicles from each group were incubated in 20 μL of lysis buffer in ice for 10 min; then, 2 μL of magnetic bead stock and 20 tetrafluoroethylene (TFE) were added to each tube. Then, sonication was performed for 15 min in a Bioruptor having each cycle of 30 s. After sonication, 0.75 μL of 0.1% formic acid was added, and heat exposure was provided to each tube for 5 min at 95 °C and placed tubes on the ice for 30 s. Then, tubes were incubated for 30 min at 45 °C in a PCR machine and supplemented with 5 µL of 400 mM iodoacetamide (IAA) and incubated for 30 min at 24 °C. The reaction was stopped by adding 5 µL of 200 mM dithiothreitol (DTT) to each tube. To purify the proteins, 1% formic acid and acetonitrile 100% (1:1) were added in 10 µL of lysate and incubated for 8 min. Then, the supernatant was removed, and the pellet was washed two times with 70% ethanol. Acetonitrile was added to the pellet and shifted to a magnetic stand. The digestion was performed by adding 10μL of trypsin and incubating at 37 °C for 16 h. In the last step, proteins were washed twice with acetonitrile, and then 10 µL of 2% dimethylsulfoxide (DMSO) and 1% formic acid were added to the tube. The digested peptides of SPF and DPF were labeled with TMT-127 (SPF) and TMT-129 (DPF), respectively, by labeling reagents according to the manufacturer’s protocol. Briefly, 41 µl of anhydrous acetonitrile (ACN) was added to 0.8 mg of tandem mass tag (TMT) labeling reagent, and then the mixture was transferred to each digested peptide sample. The solution was incubated at room temperature for 1 h, and the reaction was stopped by adding 8 mL of 5% hydroxylamine. Equal quantities of TMT-labeled peptides from both samples were taken and then combined and evaporated under vacuum.

### 2.4. Fractionation of TMT Reagent Labeled Peptides and LC–MS/MS Analyzes

Before Liquid chromatography–mass spectrometry (LC–MS/MS), labeled samples were suspended in 50 µL of buffer A (98% ddH_2_O, 2% ACN; pH 10.0) and passed through a high pH reversed-phase liquid chromatography column (RP–HPLC) (2.1 × 100 mm, 3 µm, 150 Å, C18). Buffer A and buffer B (98% ACN, 2% ddH_2_O; pH 10.0) were used for 60 min linear gradient elution (buffer B: 4–20% for 30 min, buffer B: 20–95% for 25 min, buffer B 95% for 5 min). Eluted fractions were collected every 1.5 min. A total of 20 fractions were collected. The collected fractions were desalted using a ZipTips C18 column (Millipore, Billerica, MA, USA). The processed samples were stored at −80 °C until used for mass spectrometry.

### 2.5. Peptide Identification

The desalted and dried fractions were eluted with 10 µL of solvent A (2% ACN and 0.1% formic acid). A 2 μL volume of each sample was applied to a trapping column (PepMap RSLC C18 column, 50 μm × 15 cm, 2 um nanofiber, Thermo Fisher Scientific, Bremen, Germany), applying a maximum pressure (600 bar) of 300 L/min. An analytical column (0.075 × 150 mm, 3 μm, 100 Å, Thermo Fisher Scientific, Bremen, Germany) was used for analysis. Next, we used buffer A (2% ACN and 0.1% formic acid) and buffer B (98% ACN and 0.1% formic acid). We added buffer A for 60 min gradient elution (5–40% buffer) separation 45 Minutes and buffer B (40–100%) for 10 min, and finally, 100% buffer B for 5 min to isolate the peptide. An LTQ-Orbitrap Elite Hybrid Mass Spectrometer (Thermo Fisher Scientific, Bremen, Germany) connected to an Easy-nLC 1000 nanometer liquid chromatography system (Thermo Fisher Scientific, Odense, Denmark) was used to analyze all online peptides. A data correlation model was used for mass spectrometry (MS) analysis in a scanning range of 350–1800 *m*/*z* and obtain 60,000 detection scans with a mass resolution of 400,000 *m*/*z* from an Orbitrap analyzer. In the linear ion trap, the 10 strongest precursor ions were selected for secondary mass spectrometry (MS2) analysis in high collision energy dissociation mode in the linear ion trap. The dynamic exclusion parameters included an exclusion count of two and an exclusion time of 40 s. The siloxane ion was used for internal calibration (*m*/*z* = 4,451,200). The raw proteomics data were presented in Supplementary Tables S1–S4.

### 2.6. Bioinformatics Analysis

The differential expression analysis to determine differentially expressed proteins (DEPs) was performed by using the shiny-based R program (https://infinityloop.shinyapps.io/TCC-GUI/, accessed on 15 January 2021). Gene ontology analysis of DEPs was performed by using an online database (v6.8; http://david.abcc.ncifcrf.gov/, accessed on 15 January 2021). The interactive network containing the identified DEPs was identified using the using software R. KEGG pathway analysis of DEPS was performed by using an online platform (https://www.kegg.jp/kegg/pathway.html, accessed on 15 January 2021). The DEPs are listed in Supplementary Table S4. The proteome profile of each replicate (n = 3) is listed in Supplementary Table S1–S3.

### 2.7. Validation of Proteomics Data by Using Real-Time Quantitative PCR

The proteomic data were validated using real-time quantitative PCR. For this purpose, total RNA from small primary follicles (SPFs) and developing primary follicles (DPFs) were extracted by using the Trizol method [23]. The extracted RNA was converted to cDNA by using a reverse transcription kit (6210A TaKaRa, Japan) following the manufacturer’s instructions. The primers used in the present study are presented in Table 1. The PCR cycling profile was carried out under the following conditions: 5 min at 95 °C, followed by 22 cycles of 30 s at 94 °C, 30 s at 57 °C, and 30 s at 72 °C, with a final extension of 10 min for 72 °C. The 2^−ΔΔCt^ method was used to analyze qRT-PCR data [24]. The experiment was performed in triplicate, and *β-actin* was used as a reference gene for normalization.

## 3. Results

**Figure 2:**
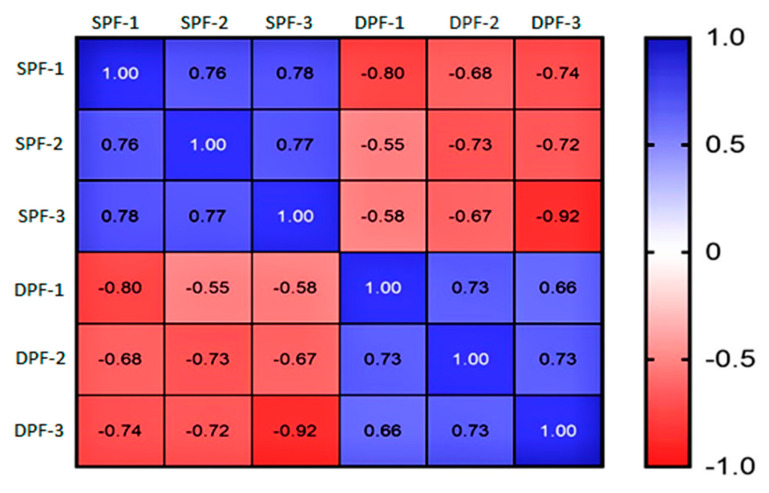
Correlation matrix. The correlation matrix is showing a higher correlation among the replicates of each experimental group, thereby validating the efficiency of proteomics data. SPFs stand for the replicates of small primary follicles and DPFs for developed primary follicles. SPFs and DPFs: Small and developing primary follicles

The present study elaborates on the proteome profile of SPFs (81–150 μm) and DPFs (300–500 μm). The precision in the proteomic was assessed by calculating the coefficient of correlation and it revealed a significant correlation between replicates of each group. The correlation matrix validated the stability of the experimental data, which can be used for subsequent analysis (Figure 2).

**Figure 3:**
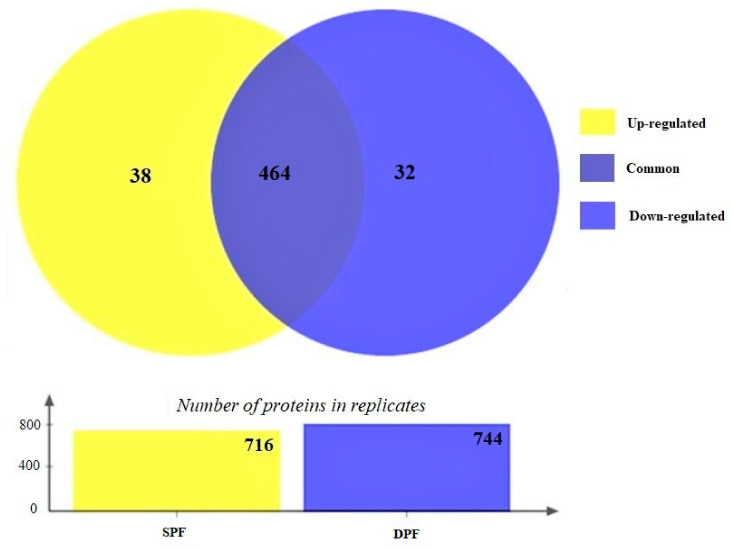
The Venn diagram illustrates a total of 464 common proteins in both experimental groups. The differentially expressed proteins were calculated based on *p*-value and fold change (*p* < 0.05 and FC ± 1.2). Based on the criteria 38 proteins were upregulated, and 32 proteins are downregulated.

The experiment was performed in three biological replicates and a total of 716 and 744 proteins were quantified in SPF and DPF groups, respectively, and 464 proteins were found to be common between both experimental groups. Figure 3 shows the Venn diagram presenting the number of identified and common proteins between both experimental groups. 

**Figure 4:**
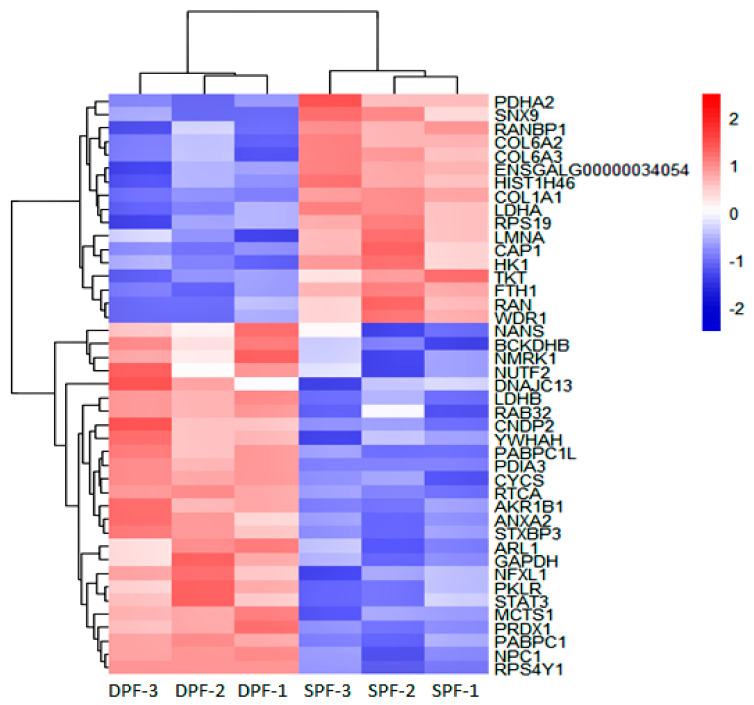
Protein profile obtained by labeled free TMT-based LC–MS/MS Analysis. Heat map showing the top DEPs and representing the distribution of DEPs, the differential proteomic (*p*-value < 0.05 and FC ± 1.2). Pink and blue bars showing proteins that are significantly up and downregulated. The SPF stands for small primary follicle and DPF for the developed primary follicle. Only the recognized proteins were displayed in the heat map.

The differential proteomic analysis (*p*-value < 0.05 and fold change (FC) ± 1.2) showed 70 DEPs (38 upregulated and 32 downregulated). Figure 4 displays the heat map showing the expression pattern of differentially expressed proteins between the replicates of experimental groups. Supplementary Table S4 shows the details about differentially expressed proteins. 

**Figure 5:**
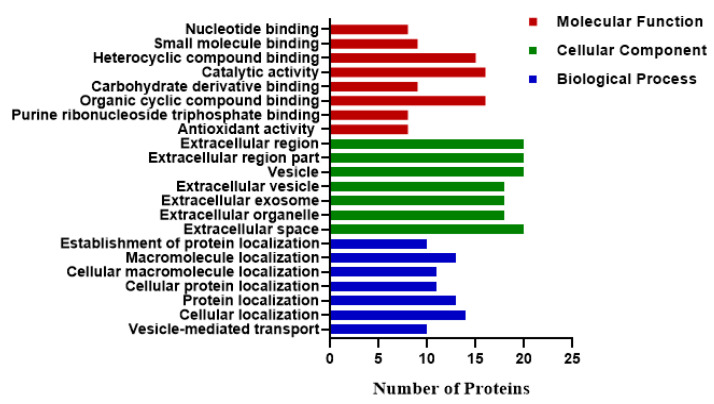
Gene ontology (GO) enrichment analysis (*p* < 0.05) of DEPs (*p*-value < 0.05 and FC ± 1.2). Proteins were classified according to their molecular functions, localization in cellular components, and participation in the biological process. The results displayed the number of proteins participating in each category.

Gene ontology enrichment analysis of the DEPs was performed to determine their role in the biological process, molecular function, and cellular component (Figure 5). Proteins involved in the cellular process were associated with extracellular matrix disassembly, extracellular vesicles, extracellular region part, and extracellular exosome. The molecular functions of DEPs included nucleotide binding, small molecule binding, carbohydrate derivative binding, and organic cyclic compound binging. The DEPs were involved in biological processes such as localization activities, including cellular protein localization, cellular macromolecule localization, macromolecule localization, and establishment of protein localization. 

**Figure 6:**
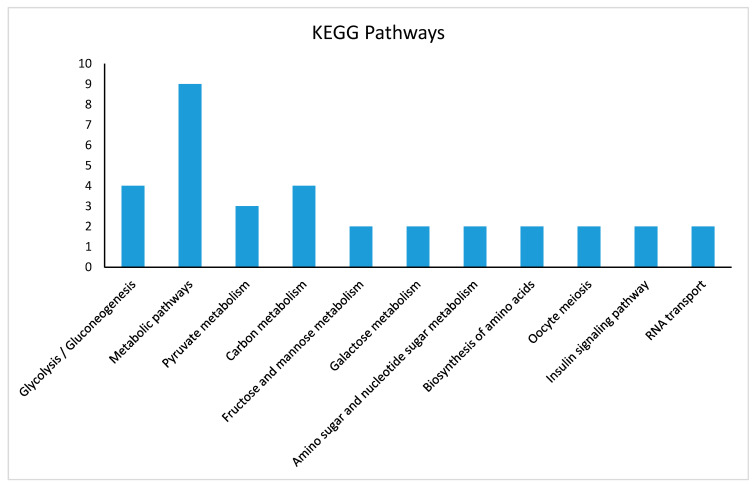
Distribution of KEGG pathway enrichment analysis (*p* < 0.05) of DEPs of small and developing primary follicles. DEPs are mainly associated with glycolysis, gluconeogenesis, carbon metabolism, pyruvate metabolism, galactose metabolism, fructose and mannose metabolism, oocyte meiosis, endocytosis, and lysosome activities. The *X*-axis represents DEPs in different pathways, and *Y*-axis represents the number of proteins involved in each KEGG pathway enrichment. KEGG: Kyoto Encyclopedia of Genes and Genomes.

The KEGG pathway enrichment analysis indicated that DEPs were associated mainly with glycolysis gluconeogenesis, carbon metabolism, pyruvate, galactose, fructose and mannose metabolism, oocyte meiosis, endocytosis, and lysosome activities. Figure 6 illustrates the distribution of proteins in various KEGG pathways.

**Figure 7:**
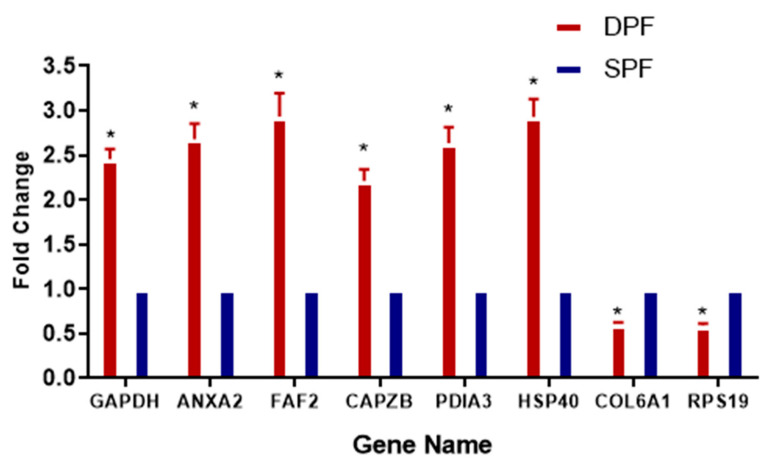
Verification of relative gene expression levels of *Gapdh*, *Anxa2*, *Faf2*, *Capzb*, *Pdia3,* and *Hsp40* and downregulated as *Col6a1*, and *Rps19* by using real-time qPCR analysis. The data are expressed as mean ± SD; and statistical significance: *p* < 0.05. The *β-actin* gene expression was used as a reference gene. Asterisk (*) indicates the statistical difference (*p* < 0.05) between the groups. SPFs and DPFs: Small and developing primary follicles

The proteomic data was validated by real time quantitative PCR analysis. The real time quantitative PCR revealed that gene expression and protein expression were having similar pattern (Figure 7). The validation of the proteomics data was performed by analyzing the expression pattern of *Anxa2*, *Capzb*, *Pdia3*, *Park7*, and *Faf2* by using real-time qPCR. The expression level of *Capzb*, *Pdia3*, *Anxa2*, *Faf2*, *Rps19*, and *Hsp40* were found to be upregulated, while *Col6a1* and *Col6a2* were found to be downregulated. The results of real-time quantitative PCR were found to be consistent with those of the LC–MS/MS.

## 4. Discussion

In chickens, follicular development involved different phases, with a follicle size ranged between 0.05 mm to more than 25 mm. The later follicular development stages such as prehierarchal follicles and preovulatory follicles are well characterized, while knowledge related to the mechanism of development and growth of primary follicles is not well documented in previous studies. The purpose of the present study was to explore mechanisms associated with primary follicle development by using a TMT-based quantitative proteomics approach. In the present study, the key role of metabolic pathways such as energy metabolism, insulin signaling pathway, and biosynthesis of amino acids during the development of primary follicles was identified. 

### 4.1. Role of Glycolysis during Primary Follicle Development

The results of the present study demonstrated glycolysis as a key energy homeostasis pathway involved in primary follicle development, compared to oxidative phosphorylation, due to higher expression of glyceraldehydehyde-3-phosphate (*Gapdh*) and pyruvate kinase (PK) and lower expression of pyruvate dehydrogenase E1 component subunit alpha (*Pdha1*). *Gapdh* is a glycolysis intermediate and converted glyceraldehyde-3-phosphate into D-glycerate 1,3-bisphosphate, in the presence of nicotinamide adenine dinucleotide (NAD+) and inorganic phosphate, and also facilitate the formation of NADH and adenosine triphosphate (ATP) [18]. In the final step of glycolysis, the pyruvate kinase enzyme converted the phosphoenolpyruvate into pyruvate with the production of ATP [25], whereas *Pdha1* is the part of the pyruvate dehydrogenase complex, which is responsible for the conversion of pyruvate into acetyl coenzyme-A [26]. 

### 4.2. Role of ANXA2, PDIA3, and CAPZB during the Primary Follicle Development

The proteomic data obtained in the present study illustrated the higher expression of annexin A2 (*Anxa2*) in large primary follicles, compared to small primary follicles. The real-time quantitative PCR analysis also validated the findings of proteomic data. Our study is in line with the previous study of Zhu et al. [12], which presented the role of *Anxa2* during follicle development. Various other studies have also been reported the key role of *Anxa2* in cell proliferation and angiogenesis in various types of tissues [27,28]. Similarly, it is also involved in cellular proliferation and angiogenesis during chicken follicle development [29], as confirmed by the findings of the present study.

Protein disulfide isomerase A3 (*Pdia3*) belongs to a family of 17 different protein disulfide isomerases (*Pdis*) capable of formation (oxidation), reduction, and rearrangement (isomerization) of the disulfide-bonding patterns of proteins and have a major role in the folding of newly synthesized proteins [30]. This is associated with matrix metalloproteinase, which plays a significant role in follicular development [13,31]. Therefore, it is suggested that higher expression of *Pdia3* in small primary follicles, compared to the developing primary follicles, might be used as a marker of primary follicle development in chicken. However, further investigations are required to elaborate on its role during the chicken follicle development. The role of *Pdia3* during follicle development could be further strengthened by the study conducted by Huo et al. [32], which revealed that in mammals, the protein disulfide isomerase may increase the secretion of follicle-stimulating hormone, which ultimately improvises the follicular development.

The proteomic data from the present study suggested that large primary follicles exhibited higher expression of F-actin-capping protein subunit beta (*Capzb*), compared to small primary follicles. The actin cytoskeleton is an essential component of various dynamic biological systems and processes [33]. Capping proteins are recognized to enhance the actin filament depolymerization and promote cell motility [34,35]. In drosophila, during organogenesis, it is involved the actin cytoskeleton organization [36]. Recently, another study has also provided evidence of *Capzb* involvement in cellular growth and motility of cancerous cells [37]. Therefore, it could be divulged from the previous and present findings that *Capzb* may be associated with the actin cytoskeleton during the development of chicken primary follicle development. 

Taken together, proteomic data depicted the expression pattern of the proteome of primary follicles during their development. Some key genes were identified, which might have their functional role in promoting the development of primary follicles, cellular proliferation, and growth. Ultimately, these proteins are suggested to take part in imparting the developmental competence of follicles to attain ovulatory capacity. These proteins may also have their role in developing the follicles from prehirarchical stage to ovulation acquisition.

## 5. Conclusions

This is the first study elaborating on the mechanism of primary follicle development in chickens. In the present study, DEPs were identified between the small primary follicles and developing primary follicles, and these DEPs were mainly involved in glycolysis, pyruvate metabolism, amino acid synthesis, and oocyte meiosis. The identified key genes including *Anxa2*, *Pdia3,* and *Capzb* might be involved in primary follicle development, and their expressions were validated further by RT-qPCR. Moreover, the present study pave a way for further functional studies of follicular development in chicken.

## Figures and Tables

**Table 1:** The primers used for real-time quantitative PCR.

Gene Name	GeneBank Accession Number	Primer Sequence (5′-0 to 3′-0)	Product Length (bp)
*Gapdh*	NM_204305.1	PF:5′-TGCCCAGAACATCATCCCA-3′ PR:5′-GCCAGCACCCGCATCAAAG-3′	295
*Pdia3*	NM_204110.3	PF:5′-GGCGCAACCGAGTTATGATG-3′ PR:5′-CTCACCCACGCTGTTGTCTA-3′	131
*Capzb*	NM_001173529.1	PF:5′-GCGACTCTTCTCCGCACATA-3′ PR:5′-AAGTCTGCACAGACCTCAGC-3′	135
*Anxa2*	XM_025153803.1	PF:5′-GAGGCAGTGATCTTGGGCTT-3′ PR:5′-CATCAGTTCCCAGGCCCTTC-3′	88
*β-actin*	NM_205518.1	PF:5′-GAGCTGAGAGTAGCCCCTGA-3′ PR:5′-CGCACAATTTCTCTCTCGGC-3′	353
*Hsp40*	XM_015278955.2	PF:5′-AGGCTCTGCTTCCTCCAAGA-3′ PR:5′-ACGGTAGGTTTGCTCGCTTG-3′	95
*Faf2*	XM_414548.6	PF:5′-GTCGGGTTACTGACCCAGTG-3′ PR:5′-AGTGCCTGGCTGTAAGTTCC-3′	109
*Col6a1*	NM_205107.1	PF:5′-CCTCGTGGCGCAAGTTAAAG-3′ PR:5′-TCTCCTTGAGATGGGAGCCA-3′	278
*Rps19*	XM_025146195.1	PF:5′-CAAGCTGAAGGTTCCGGACT-3′ PR:5′-ATCTTCGTCATGGAGCCCAC-3′	156

## Data Availability

Not applicable.

## Supplementary Materials

The following are available online at https://www.mdpi.com/article/10.3390/ani11041108/s1, Table S1: Biological replicate 1, Table S2: Biological replicate 2, Table S3: Biological replicate 3, Table S4: List of differentially expressed proteins.
