# Supplementary material for: Proteomic Analysis Identifies Potential Markers for Chicken Primary Follicle Development
Source: Animals (Basel). 2021 Apr 13;11(4):1108. doi: 10.3390/ani11041108 (PMC8069082; doi:10.3390/ani11041108)
Supplement: Supplementary file 1 [file animals-11-01108-s001.zip › Table-S4 (List of Differentially Expressed Proteins.docx]

**List of Differentially Expressed Proteins**

| **Name** | **Description** | **P-Value** | **FC** |
| --- | --- | --- | --- |
| A0A1D5NTF3 | Fas associated factor family member 2 OS=Gallus gallus OX=9031 GN=FAF2 PE=4 SV=1 - [A0A1D5NTF3_CHICK] | 0.028116 | 3.016393 |
| F6RS71 | Plastin-2 OS=Gallus gallus OX=9031 GN=LCP1 PE=4 SV=1 - [F6RS71_CHICK] | 0.023739 | 2.823944 |
| P17785 | Annexin A2 OS=Gallus gallus OX=9031 GN=ANXA2 PE=1 SV=2 - [ANXA2_CHICK] | 0.020679 | 2.638158 |
| Q5ZI42 | Malignant T-cell-amplified sequence 1 OS=Gallus gallus OX=9031 GN=MCTS1 PE=2 SV=1 - [MCTS1_CHICK] | 0.025338 | 2.258242 |
| A0A1D5PXW2 | Eukaryotic translation initiation factor 3 subunit M OS=Gallus gallus OX=9031 GN=EIF3ML PE=4 SV=1 - [A0A1D5PXW2_CHICK] | 0.016902 | 2.098958 |
| F1NT57 | Aldo-keto reductase family 1, member B10 (aldose reductase) OS=Gallus gallus OX=9031 GN=AKR1B10L1 PE=4 SV=2 - [F1NT57_CHICK] | 0.000685 | 2.02139 |
| E1C8Z9 | DAB2, clathrin adaptor protein OS=Gallus gallus OX=9031 GN=DAB2 PE=4 SV=4 - [E1C8Z9_CHICK] | 0.013928 | 2.013393 |
| E1BTS5 | Syntaxin-binding protein 3 OS=Gallus gallus OX=9031 GN=STXBP3 PE=3 SV=3 - [E1BTS5_CHICK] | 0.00864 | 1.875576 |
| E1BWJ7 | Polyadenylate-binding protein OS=Gallus gallus OX=9031 GN=PABPC1L PE=3 SV=3 - [E1BWJ7_CHICK] | 0.006541 | 1.845133 |
| P67881 | Cytochrome c OS=Gallus gallus OX=9031 GN=CYC PE=1 SV=2 - [CYC_CHICK] | 0.010446 | 1.821596 |
| Q49B65 | EF hand-containing protein 1 OS=Gallus gallus OX=9031 GN=EFHD1 PE=2 SV=1 - [Q49B65_CHICK] | 0.045615 | 1.80531 |
| Q5ZKJ2 | Tyrosine 3-monooxygenase/tryptophan 5-monooxygenase activation protein eta OS=Gallus gallus OX=9031 GN=YWHAH PE=2 SV=1 - [Q5ZKJ2_CHICK] | 0.025152 | 1.773196 |
| A0A1D5P765 | 2-oxoisovalerate dehydrogenase subunit beta, mitochondrial precursor OS=Gallus gallus OX=9031 GN=BCKDHB PE=4 SV=1 - [A0A1D5P765_CHICK] | 0.027224 | 1.6875 |
| A0A1I7Q414 | Actin, alpha skeletal muscle OS=Gallus gallus OX=9031 GN=ACTA1 PE=3 SV=1 - [A0A1I7Q414_CHICK] | 0.042386 | 1.684211 |
| A0A1L1RN46 | Nuclear transport factor 2 OS=Gallus gallus OX=9031 GN=NUTF2 PE=4 SV=1 - [A0A1L1RN46_CHICK] | 0.03016 | 1.614035 |
| Q6DV79 | Signal transducer and activator of transcription 3 OS=Gallus gallus OX=9031 GN=STAT3 PE=1 SV=2 - [STAT3_CHICK] | 0.04469 | 1.572016 |
| P0CB50 | Peroxiredoxin-1 OS=Gallus gallus OX=9031 GN=PRDX1 PE=1 SV=1 - [PRDX1_CHICK] | 0.010868 | 1.528926 |
| A0A1L1RVT1 | Creatine kinase B-type OS=Gallus gallus OX=9031 GN=CKB PE=3 SV=1 - [A0A1L1RVT1_CHICK] | 0.01162 | 1.46888 |
| A0A1D5NZ71 | DnaJ heat shock protein family (Hsp40) member C13 OS=Gallus gallus OX=9031 GN=DNAJC13 PE=4 SV=1 - [A0A1D5NZ71_CHICK] | 0.009852 | 1.458333 |
| E1C350 | Nicotinamide riboside kinase 1 OS=Gallus gallus OX=9031 GN=NMRK1 PE=4 SV=1 - [E1C350_CHICK] | 0.001035 | 1.455285 |
| F1NQT4 | NPC intracellular cholesterol transporter 1 OS=Gallus gallus OX=9031 GN=NPC1 PE=4 SV=3 - [F1NQT4_CHICK] | 0.008366 | 1.45339 |
| F1P463 | Cytosolic non-specific dipeptidase OS=Gallus gallus OX=9031 GN=CNDP2 PE=4 SV=1 - [F1P463_CHICK] | 0.037932 | 1.453125 |
| A0A1L1RMP9 | ADP ribosylation factor like GTPase 1 OS=Gallus gallus OX=9031 GN=ARL1 PE=3 SV=1 - [A0A1L1RMP9_CHICK] | 0.02228 | 1.376984 |
| E1C027 | Nuclear transcription factor, X-box binding like 1 OS=Gallus gallus OX=9031 GN=NFXL1 PE=4 SV=3 - [E1C027_CHICK] | 0.013567 | 1.372624 |
| F1NBL3 | RAB32, member RAS oncogene family OS=Gallus gallus OX=9031 GN=RAB32 PE=4 SV=3 - [F1NBL3_CHICK] | 0.045803 | 1.363636 |
| A0A1L1RWJ7 | Phospholipase A2 activating protein OS=Gallus gallus OX=9031 GN=PLAA PE=4 SV=1 - [A0A1L1RWJ7_CHICK] | 0.046641 | 1.354582 |
| P00356 | Glyceraldehyde-3-phosphate dehydrogenase OS=Gallus gallus OX=9031 GN=GAPDH PE=2 SV=3 - [G3P_CHICK] | 0.04195 | 1.350943 |
| A0A1L1RUZ7 | O-acyl-ADP-ribose deacylase 1 OS=Gallus gallus OX=9031 GN=OARD1 PE=4 SV=1 - [A0A1L1RUZ7_CHICK] | 0.042061 | 1.323651 |
| A0A1D5PD02 | Ubiquitin like modifier activating enzyme 7 OS=Gallus gallus OX=9031 GN=UBA7 PE=3 SV=1 - [A0A1D5PD02_CHICK] | 0.009397 | 1.31746 |
| Q5ZMH8 | N-acetylneuraminate synthase OS=Gallus gallus OX=9031 GN=NANS PE=2 SV=1 - [Q5ZMH8_CHICK] | 0.021704 | 1.304348 |
| P00337 | L-lactate dehydrogenase B chain OS=Gallus gallus OX=9031 GN=LDHB PE=1 SV=3 - [LDHB_CHICK] | 0.020801 | 1.296875 |
| E1C1Y0 | Uncharacterized protein OS=Gallus gallus OX=9031 PE=4 SV=3 - [E1C1Y0_CHICK] | 0.0043 | 1.279412 |
| R4GJD6 | Eukaryotic translation initiation factor 4E family member 1B OS=Gallus gallus OX=9031 GN=EIF4E1B PE=3 SV=3 - [R4GJD6_CHICK] | 0.025106 | 1.274436 |
| P47836 | 40S ribosomal protein S4 OS=Gallus gallus OX=9031 GN=RPS4 PE=2 SV=2 - [RS4_CHICK] | 0.004206 | 1.2607 |
| A0A1D5NYB2 | Polyadenylate-binding protein OS=Gallus gallus OX=9031 GN=PABPC1 PE=3 SV=1 - [A0A1D5NYB2_CHICK] | 0.014744 | 1.243151 |
| A0A1L1RUA9 | F-actin-capping protein subunit beta isoforms 1 and 2 OS=Gallus gallus OX=9031 GN=CAPZB PE=4 SV=1 - [A0A1L1RUA9_CHICK] | 0.034016 | 1.216606 |
| Q8JG64 | Protein disulfide-isomerase A3 OS=Gallus gallus OX=9031 GN=PDIA3 PE=2 SV=1 - [PDIA3_CHICK] | 0.003317 | 1.214286 |
| F1NW43 | Pyruvate kinase OS=Gallus gallus OX=9031 GN=PKM PE=3 SV=2 - [F1NW43_CHICK] | 0.048285 | 1.202091 |
| A0A1D5PYU1 | Uncharacterized protein OS=Gallus gallus OX=9031 PE=4 SV=1 - [A0A1D5PYU1_CHICK] | 2.91E-05 | 0.322785 |
| F1NDN6 | Keratin 12 OS=Gallus gallus OX=9031 GN=KRT12 PE=3 SV=3 - [F1NDN6_CHICK] | 0.039271 | 0.435798 |
| A0A1L1RQY9 | 60S acidic ribosomal protein P1 OS=Gallus gallus OX=9031 GN=RPLP1 PE=3 SV=1 - [A0A1L1RQY9_CHICK] | 0.034986 | 0.454545 |
| A0A1D5PMQ5 | Uncharacterized protein OS=Gallus gallus OX=9031 GN=KRTC42L PE=3 SV=2 - [A0A1D5PMQ5_CHICK] | 0.007706 | 0.496016 |
| Q5ZJ38 | Uncharacterized protein OS=Gallus gallus OX=9031 GN=RANBP1 PE=2 SV=1 - [Q5ZJ38_CHICK] | 0.031988 | 0.496454 |
| E1BTY4 | Sorting nexin OS=Gallus gallus OX=9031 GN=SNX9 PE=3 SV=2 - [E1BTY4_CHICK] | 0.040814 | 0.507726 |
| A0A1D5NW11 | Keratin, type I cytoskeletal 19 OS=Gallus gallus OX=9031 GN=KRT19 PE=3 SV=1 - [A0A1D5NW11_CHICK] | 0.040275 | 0.543182 |
| A0A1D5PTH0 | Esterase D OS=Gallus gallus OX=9031 GN=ESD PE=4 SV=1 - [A0A1D5PTH0_CHICK] | 0.009456 | 0.560091 |
| A0A1L1RXA4 | F-actin-capping protein subunit alpha-2 OS=Gallus gallus OX=9031 GN=CAPZA2 PE=3 SV=1 - [A0A1L1RXA4_CHICK] | 0.039012 | 0.62069 |
| A0A1D5PS16 | WD repeat-containing protein 1 OS=Gallus gallus OX=9031 GN=WDR1 PE=4 SV=1 - [A0A1D5PS16_CHICK] | 0.032485 | 0.650131 |
| A0A1D5PLK4 | Collagen alpha-2(VI) chain OS=Gallus gallus OX=9031 GN=COL6A2 PE=4 SV=1 - [A0A1D5PLK4_CHICK] | 0.027934 | 0.660221 |
| Q00649 | Heat shock protein beta-1 OS=Gallus gallus OX=9031 GN=HSPB1 PE=2 SV=1 - [HSPB1_CHICK] | 0.001612 | 0.677054 |
| P08267 | Ferritin heavy chain OS=Gallus gallus OX=9031 GN=FTH PE=2 SV=2 - [FRIH_CHICK] | 0.019575 | 0.677419 |
| P20763 | Ig lambda chain C region OS=Gallus gallus OX=9031 PE=4 SV=1 - [LAC_CHICK] | 0.026046 | 0.703264 |
| F1NP51 | Lamin-B2 OS=Gallus gallus OX=9031 GN=LMNB2 PE=3 SV=2 - [F1NP51_CHICK] | 0.033614 | 0.70977 |
| E1BRE9 | Decorin OS=Gallus gallus OX=9031 GN=DCN PE=3 SV=2 - [E1BRE9_CHICK] | 0.017139 | 0.714286 |
| E1BTT8 | L-lactate dehydrogenase OS=Gallus gallus OX=9031 GN=LDHA PE=3 SV=2 - [E1BTT8_CHICK] | 0.002442 | 0.716292 |
| A0A1D5Q032 | Adenylyl cyclase-associated protein OS=Gallus gallus OX=9031 GN=CAP1 PE=3 SV=1 - [A0A1D5Q032_CHICK] | 0.027153 | 0.719101 |
| P62801 | Histone H4 OS=Gallus gallus OX=9031 GN=H4-I PE=1 SV=2 - [H4_CHICK] | 0.00864 | 0.723837 |
| A0A1L1RMV9 | Ubiquinol-cytochrome c reductase core protein 2 OS=Gallus gallus OX=9031 GN=UQCRC2 PE=4 SV=1 - [A0A1L1RMV9_CHICK] | 0.027984 | 0.749263 |
| F1NZJ2 | Hexokinase-1 OS=Gallus gallus OX=9031 GN=HK1 PE=3 SV=3 - [F1NZJ2_CHICK] | 0.030447 | 0.75 |
| Q5F426 | Pyruvate dehydrogenase E1 component subunit alpha OS=Gallus gallus OX=9031 GN=PDHA1 PE=2 SV=1 - [Q5F426_CHICK] | 0.014139 | 0.751381 |
| F1P1A5 | Transketolase OS=Gallus gallus OX=9031 GN=TKT PE=4 SV=2 - [F1P1A5_CHICK] | 0.044763 | 0.751429 |
| P20785 | Collagen alpha-1(VI) chain OS=Gallus gallus OX=9031 GN=COL6A1 PE=2 SV=1 - [CO6A1_CHICK] | 0.031044 | 0.766667 |
| A0A1L1RR33 | Tubulin alpha chain OS=Gallus gallus OX=9031 GN=TUBA3E PE=3 SV=1 - [A0A1L1RR33_CHICK] | 0.00682 | 0.767647 |
| A0A1L1RM44 | DEAD-box helicase 4 OS=Gallus gallus OX=9031 GN=DDX4 PE=3 SV=1 - [A0A1L1RM44_CHICK] | 0.043681 | 0.768072 |
| A0A1D5PAQ8 | Nuclear migration protein nudC OS=Gallus gallus OX=9031 GN=NUDC PE=4 SV=1 - [A0A1D5PAQ8_CHICK] | 0.018386 | 0.77208 |
| A0A1D5PDV6 | Ribosomal protein S19 OS=Gallus gallus OX=9031 GN=RPS19 PE=4 SV=1 - [A0A1D5PDV6_CHICK] | 0.007973 | 0.773994 |
| A0A1B1XXT0 | Eukaryotic translation initiation factor 5A OS=Gallus gallus OX=9031 GN=EIF5A2 PE=2 SV=1 - [A0A1B1XXT0_CHICK] | 0.021903 | 0.77439 |
| P42558 | GTP-binding nuclear protein Ran OS=Gallus gallus OX=9031 GN=RAN PE=2 SV=1 - [RAN_CHICK] | 0.049414 | 0.776786 |
| F1NY46 | Uncharacterized protein OS=Gallus gallus OX=9031 GN=CPEB1 PE=4 SV=3 - [F1NY46_CHICK] | 0.013527 | 0.779037 |
| A0A1D5PGD5 | Collagen alpha-3(VI) chain OS=Gallus gallus OX=9031 GN=COL6A3 PE=4 SV=1 - [A0A1D5PGD5_CHICK] | 0.022184 | 0.79697 |
